# Insider apology for microeconomic theorising?

**DOI:** 10.1080/1350178X.2024.2326895

**Published:** 2024-03-18

**Authors:** Maarten Janssen, Tarja Knuuttila, Mary S. Morgan

**Affiliations:** a Department of Economics, University of Vienna, Vienna, Austria; b Department of Philosophy, University of Vienna, Vienna, Austria; c Department of Economic History, London School of Economics, London, England

**Keywords:** Theories, models, economics, microeconomics, interpretation

## Abstract

This comment on 'Economic theories and their Dueling interpretations' questions the descriptive adequacy of the ‘sociology of economics' proposed by Gilboa, Postlewaite, Samuelson, and Schmeidler (GPSS) (2022). We ask whether economists still perceive the role of microeconomic theory as central as do GPSS. In particular, is present-day economics unified by the principles of maximising, subject to constraints and equilibrium analysis? We argue that this is not the case. GPSS’ appeal to the interpretative flexibility of economic theories appears apologetic, especially the suggestion that theories and models, which once were considered positive descriptions or predictive instruments, are now cast as analytical or methodological exercises. We conclude on a more constructive note, drawing from the recent philosophical discussion of modelling which, quite paradoxically, grants highly idealized and simplified models a more important role than GPSS appear to allow.

## 1. Introduction

The article by Gilboa, Postlewaite, Samuelson, and Schmeidler (from here on collectively referred to as GPSS) (2022) makes for an interesting read. It is not so often that economists who have made well-known contributions to their field reflect on their own discipline. The authors claim to contribute to the ‘sociology of economics’ by providing an overview of the very different ways that economists interpret their theories and theoretical models. Upon closer inspection, however, we were puzzled by how exactly one is meant to read the article. First, there is the question of descriptive adequacy: is this how economists more generally view their discipline nowadays, or is this the view that these four microeconomic theorists have about how the role of economic theory should be understood? Second, and related to the first question, is present economic theorising unified by the principles of maximising, subject to constraints and equilibrium analysis? Furthermore, to the extent that economic models do embody such a unifying framework: what epistemic work does it do for economists? Third, one cannot escape the feeling that the authors’ appeal to the interpretative flexibility of economic theories is largely apologetic in nature. They suggest that theories and models that once were considered positive descriptions of real economic behaviour, or genuinely predictive instruments, should now be considered as amounting to mere analytical or methodological exercises. Fourth, even though GPSS explicitly claim that the nature of their analysis is positive, and state ‘[o]ur goal is to model the way that economists think about their models’ (GPSS, p. 2), they also suggest, in the same stroke, rationales for ‘why economists are so blasé about observations that their assumptions are far-fetched, and so unperturbed by refutations of their theories’ (GPSS, p. 2). In other words, by describing different possible interpretations of economic theories, GPSS suggest that there are interpretations that legitimise economic models that would otherwise have been refuted.

We will organise our commentary around these four points, concluding on a more constructive note. We draw from recent philosophical discussions of modelling which, quite paradoxically, grant highly idealized and simplified models a more important role than GPSS seem to allow. We consider their implicit criteria for a ‘positive’ role of models unduly stringent in the sense that they presume that if the assumptions of a model are unrealistic, the model cannot provide understanding of the economy but rather can only do so regarding economic theorising, or the methodology of economics. This need not be the case. Indeed, the suggestion of GPSS to consider economic models as theoretical cases points towards their ability to provide understanding of various *kinds of* economic situations and the modal role of models as an exploration of different possibilities. We also observe that economics has been problem-led for the last 15 odd years or more, seeking to understand the problems and questions of e.g. poverty, inequality, how markets really work, how institutions are designed, and the economic side of the climate problem. By focussing on alternative ways to interpret economic models, GPSS do not do justice to this development and focus mainly on how to rationalize theories that have been developed before this change in focus. What is more, much economics-inspired theoretical modelling is now being done in applied social science domains.

## 2. The current state of economics

The authors claim a sociology of science perspective of economics: they want to understand how economists themselves interpret the models they work with. They base their article on their own ‘life among the econs’, the many conversations they have had with other economists, and the work they have done as editors of important journals in economics. Given this, it may seem difficult to argue with their account: after all, they have spent their working lives in the field. However, taking a cursory look at the very best academic journals in economics (the ‘Top 5’), attending some of the yearly conferences in economics or some of the many seminars, and reading the rumours around the economics job market (e.g. through https://www.econjobrumors.com/), one may get the feeling that the perspective that the authors are providing is somewhat outdated. Economics has shifted quite substantially away from purely theoretical modelling towards more applied theory approaches (theory being ‘applied’ to analyse all kinds of problems and situations – as discussed in section 3 below) and towards much more data-driven approaches coming out of the ‘experimental’ frameworks of lab, field, and ‘natural’ experiments (discussed immediately below). We have not performed a rigourous analysis of whether or not this impression is correct, as for that one should perform a comparative analysis of say, the economics of 50 years ago and the current state of economics, but some observations point in this direction.

First, over the last 5 years, two Nobel prizes have been given to research in which the role of economic theory is rather limited. In 2019, the prize was awarded to Banerjee, Duflo, and Kremer for their ‘experimental approach to alleviating global poverty’.^1^ This prize was essentially a Nobel Prize for doing applied economics where the methodology is to apply random controlled trial (RCT) experiments in economics and then analyse the results econometrically; many studies especially in development economics use this methodology. In 2021, the prize was awarded to Angrist, Card, and Imbens ‘for their methodological contributions to the analysis of causal relationships’.^2^ The regression discontinuity approaches that these authors developed and/or applied essentially use natural policy experiments to try to uncover causal relationships using statistical data analysis. In both areas, the role of economic theory is extremely limited. GPSS ignore these prizes in their article. In discussing machine learning (ML) techniques, they associate RCT methodologies with fields such as medicine and ask the question, ‘who needs to theorize if we can predict better using theory-free, general-purpose techniques?’ They answer that ‘economists often respond that they seek causal relationships, which are particularly important for policy questions, and therefore they cannot abandon their theories in favour of ML techniques’ (GPSS, p. 7). In other words, they subscribe to a particular kind of causal analysis, derived from theoretical economic modelling (though considering some of their subsequent interpretations of economic modelling – see the discussion below – they appear to commit to a view that many, if not most, economic models fall short of supplying causal explanations).

Second, if one opens the pages of journals such as the *American Economic Review*, the *Journal of Political Economy,* or *The Quarterly Journal of Economics,* one is struck by the number of papers that do not appear to be based on any theory at all. An applied, or policy question is posed, the dataset that is used is extensively discussed together with the institutional background, a statistical model is introduced and often some type of instrumental variable approach and diff-in-diff techniques are employed to try to uncover causal relationships. Again, without having performed a proper empirical analysis, our impression is that within the number of papers published, also in the top 5 journals, the portion of advanced microeconomic theory is declining. Not to mention that the two Nobel prizes mentioned above seem to have powerful followers in the economics profession.

Third, one clearly gets the impression that the number of jobs that are available in the junior market for micro theory is declining as well and that the competition among candidates in this field is much stronger (as there is relatively little demand) in comparison to other fields of economics. Many economics departments around the world appear keener on hiring applied people who can work with data and teach students applied econometrics than hiring microeconomic theorists.

The above observations cast doubt on economics still being the same as the economics we know of the time of Arrow, Aumann, Savage, and the other economists GPSS write much about. While they suggest that economics has not changed much in view of the wider availability of large data sets, it appears to us that economics has in fact changed and that it has moved towards areas in which economic theory does not play much of an obvious role (such as in the research that was awarded with the Nobel prize in 2019 and 2021), or indeed any at all. We do not want to engage in a normative assessment of whether this development is for the good of the field. However, given that GPSS take themselves to be doing ‘sociology of science’, it should be an interesting question of whether our rather opposite impression, i.e. that there is a shift towards less theory-laden economics, is correct.

In response to these points, GPSS may say that their article describes the view of economic theorists and not of the profession as a whole. They may well be right on this account, but that view itself may be less relevant if the role of theorists in the profession is declining.

## 3. A unifying framework?

GPSS begin their article as follows: ‘Among the social sciences, economic theory is arguably unique in embracing a single, unifying conceptual framework. Most research in economic theory assumes that each agent maximizes an objective function subject to constraints, and the analyst then focuses on the equilibria defined by the interaction of such agents’ (GPSS, p. 1). In the previous section, we argued that many applied fields in economics are no longer based on these theoretical premises. GPSS appear to be correct, however, that at least most research in economic theory itself shares the principles of maximisation subject to constraints and equilibrium analysis. The question we want to raise here is to what extent this can be viewed as a ‘unifying framework’ as GPSS claim.

If the principles of constrained optimisation and equilibrium are important, unifying elements, they should have an important role to play in any model that aims to have explanatory or predictive power. That is, the ‘explanation’ that is given with the model must in some sense depend on these principles and that, if the model were to be stripped of these principles (i.e. the models would no longer embed or embody them), the explanation would not be the same. However, we do not think that these principles play such a critical role in economic theories that aim to explain or predict. To illustrate, consider two classic models of economic theory that have also been discussed in the methodology of economics:^3^ (i) the Akerlof lemons model and (ii) Thomas Schelling’s checkerboard model.

The main claim of Akerlof (1970) is that in markets where there is asymmetric information between buyers and sellers, good quality products may not be traded as the buyers’ willingness to pay for average quality can be lower than the minimum price sellers want to get for high-quality products. Moreover, Akerlof points out that the market may react to such information asymmetries by generating ‘institutions’ such as guarantees to overcome this ‘lemons’ problem. That is, the existence of such institutions as guarantees can in many actual markets be ‘explained’ by the existence of information asymmetries. The main claim of Schelling (1971, 1978), in turn, is that segregation can arise even if people do not have strong preferences for segregation. Instead, within the Schelling model, the interaction between individuals is such that segregation is an unintended consequence of the decisions that individuals (with weak preferences) make given the particular neighbourhoods they find themselves in. To be sure, Schelling’s model may not appear to be an economic model, especially by the criteria spelled out by GPSS. It nevertheless provides a potential invisible-hand explanation of segregation in relying on the unintentional consequences of intentional actions.

As for the supposed unification provided by the principles of maximising, subject to constraints and equilibrium analysis, it is important to note that in the above descriptions of the lemons model and the checkerboard model, the words ‘optimisation and ‘equilibrium’ do not play any crucial role. It is true that in Akerlof (1970), individuals do optimise an objective function and an equilibrium analysis is performed, but these principles are used more in terms of ‘even if’ than ‘because of’. That is, the lemons principle holds *even if* individuals optimise: it is not the case that the lemons principle holds *because* individuals optimise. The same holds true for Schelling’s model (1971, 1978), where it is possibly even more evident that individual optimisation does not play a role, as Schelling appears to argue that irrespective of the precise objective function, segregation arises.

Thus, in so far as economics still uses theoretical models (though in the previous section we have argued that this may be less so), these two theoretical models of Schelling and Akerlof appear to be more in line with what Popper (1957), Latsis (1972), or Koertge (1975) have approached as capturing the logic of a situation: outcomes in the social sciences, which are to be understood from the actions social and economic actors take given the kind of situation they find themselves in. (E.g. Akerlof’s model captures and applies to a generic kind of situation (one of asymmetric information), not a fully specified individual situation nor to a general case, but to something in between.) So, the emphasis here is more on the contextualisation into types of situations, than on what drives individual agents. In other words, situational logic is a much broader category than that given by principles of maximisation and equilibrium analysis, as it emphasises the constraints and other features of the situation, rather than whether or not the agent is maximising something. As Morgan (2012) has pointed out: characterising situations as kinds or types gives such models some explanatory power even if the rationality principles do little (see also Cartwright, 1999). A lot of game theory (in so far as it tries to explain or predict certain real-world observations) can be understood in this way: it is the rules of the game that to a large extent drive the outcome, rather than what exactly drives individual agents that are placed within that kind of context. Unlike the second principle of Newtonian mechanics, individual optimisation and equilibrium are (more or less) empty shelves. The reason why economic theorists use them lies elsewhere and not so much in their unifying power. For example, optimisation subject to constraints may be a mathematically convenient way to model incentives, and an equilibrium situation is then one where these incentives are at least consistent with each other. But these principles do not provide any explanatory power, rather that power comes from the model-based analysis of the setting in which agents operate.

## 4. Interpretative flexibility: from positive to analytical interpretations

At the outset, there is not anything that spectacular about the claim that economic models can be interpreted in different ways – and that they have various uses. Nothing could be more commonplace when it comes to contemporary modelling practice. Models are used to explain, predict, design experiments, probe theories, and explore theoretical and other possibilities (e.g. Gelfert, 2016; Morgan, 2012; Weisberg, 2013). Moreover, they provide research objects of their own: their properties are studied, and they supply templates for further modelling, often transferable to study some other problems or targets than the model was originally designed to address. Apart from analysing the multiplex nature of models and the various ways they function as epistemic tools and representations, the present philosophical discussion of models has documented multiple modelling practices across different disciplines (Knuuttila et al., forthcoming; e.g. Morgan & Morrison, 1999). So, what’s at stake in the claim that economic models can be interpreted in many ways?

The first thing to note is that GPSS’s endeavour to argue for the interpretative flexibility of economic models is motivated by two perceived problems of economics. In the introductory section of their article, they observe that economics ‘has been under persistent attack for failing to produce accurate predictions’ and that there have been calls for economics to ‘quit the game of making implausible assumptions and deriving unrealistic conclusions’ (GPSS, p. 1). Given the apparently insoluble problems of insufficient predictive power and unrealistic assumptions, the interpretative flexibility of economic models comes to the rescue.

GPSS approach the interpretative flexibility of economic models through five different distinctions that propose different ways in which the criticisms concerning the insufficient predictive power and unrealistic assumptions and results could be kept at bay. They start their discussion from the distinction between positive, normative, and analytical models in terms of their usage or function for economists.^4^ That GPSS talk about different kinds of models throughout their paper is somewhat confusing, since what they actually argue for is that the very same model can afford different interpretations. Their focus throughout the paper is mostly on the analytical interpretation of models, that is, models being used for analysis of a theory or its assumptions, subsequently serving an analytical purpose. They claim that ‘[t]he positive and normative categories have garnered the lion’s share of attention’ while the analytical interpretation is ‘often not mentioned’ (GPSS, p. 4). Their analytical interpretation of economic models overlaps with their idea of viewing models in terms of economic methodology which is introduced later in the article.^5^

Addressing the ‘duelling interpretations’ of economic models, GPSS discuss examples of economic models that were *originally* either taken as ‘positive models’ but are not anymore (e.g. the ‘classical’ view of game theory), or could be taken as such only under very special circumstances that have not, and perhaps could not, ever be realised (e.g. Arrow’s impossibility theory), or were not even at the outset apt for a ‘positive’/ realistic interpretation (e.g. Aumann, 1987 model of Bayesian rationality in games). According to GPSS, these and similar cases are best interpreted as ‘analytical models’, especially ‘when facing aggressive audience’, in which case the economist(s) ‘might step back and rather than promoting a model as an explanation of a real-life phenomenon, present it as a ‘proof of concept’’ or ‘merely an exercise in testing the scope of the standard paradigm’ (GPSS, p. 7). One cannot but wonder whether the economist(s) would step back when facing other kinds of audiences.

Taking GPSS at face value, the question is whether, under an analytical interpretation, a model can have any other value than a methodological one, or one that only concerns economic theorising itself? GPSS do indeed suggest another strategy for defending economic models: considering them in terms of critique. In their view, if the modelling framework can accommodate some stylized economic facts, the burden of proof is shifted onto the challengers of economic theory. It is hard to see, however, how an analytically interpreted model would have such a force, unless it possessed some initial credibility (e.g. in the sense of Sugden, 2000). Why would the critics of economic modelling accept the terms of some model or modelling framework when it was their unrealistic assumptions and results that were criticised in the first place? It is not enough that a particular model can render some stylized facts. Apart from such facts being merely stylized, the usual situation faced by the scientists and public alike is that of theoretical underdetermination: either the available evidence is insufficient, or there are empirically more or less equivalent alternatives.

Consequently, for analytically interpreted models to function as a means for critique, they would need to be empirically supported, and not just serve as devices for analytical purposes. And GPSS do indeed attribute some empirical backing also to such models, in addition to their more analytical roles, such as conceptual inquiry, testing implications of theoretical axioms, and the consistency of the model vis-à-vis the paradigm. The consistency checks that GPSS for the most part discuss do, not only refer to stylized facts but also, somewhat more strongly, to the consistency of ‘standard assumptions with an observed phenomenon’ (GPSS, p. 11) and the ‘consistency of a theory with a given phenomenon’ (GPSS, p. 6).

GPSS’s discussion of explanation and analogical reasoning testifies to the same kind of gesture of also reading empirical content into analytical and methodological interpretations.^6^ They argue that one should consider economic models as theoretical cases that are applied by ‘analogical reasoning’ to different domains.^7^ In philosophical discussions, explanations are often considered causal, but GPSS underline, interestingly, the importance of models being able to address new problems. They invoke also multiple modelling, as they think that reformulations of the same theory help modelers understand how a theory developed in one domain can be transferred to another.^8^ The question then becomes this: to which situations does the conception of models-as-theoretical-cases apply? GPSS argue that while the original ‘classical’ view of game theory considered game theoretic models as literal descriptions of interactions, they should more properly be viewed as potential analogies, that is, theoretical cases that may ‘capture some elements of a strategic interaction while excluding others.’ Consequently, they appear to suggest that such models are informative about some aspects of select possible real-world situations. Later in the article, they discuss Akerlof’s (1970) lemons model and Friedman’s (1971) study of collusion in oligopoly as methodological contributions, yet claim that these papers ‘appeared to capture something essential’ about their intended applications. Therefore, the question does not seem be one of interpreting the same model either as an analytical/methodological or a positive contribution, but of the model often being all of these things at once!

All things considered, given their multidimensional reading of economic models, the interpretative flexibility in the hands of GPSS appears to boil down to flexibility in catering to different audiences: depending on the expected reception, the economist can solicit different interpretations. For aggressive audiences, an economist can serve analytical or methodological interpretations, or even pose them the challenge of coming up with better explanations themselves. In front of more welcoming audiences, the economist does not need to invoke a defensive strategy, casting economic models in more positive terms.

## 5. The justificatory aim

Despite their outspoken goal of doing sociology of science, the thrust of GPSS argument is justificatory. By describing the different ways economic models are interpreted by practitioners, the authors aim to give justification for microeconomic theorising. After describing what theoretical behavioural economics has (not) achieved and the puzzles this leads to, the authors ask: ‘why are economists so content with their theories?’ and set out to explore ‘other ways in which economists find their theories and results valuable, in the hope of better understanding these puzzles’ (GPSS p. 2). Clearly, by better explaining the various ways that economists interpret their models, GPSS aim to convey the idea that also non-economists should realise that economic theory is useful. One may indeed be convinced that the more possible interpretations of economic models there are, the more likely it is that a model is useful according to at least one, but possibly on several accounts.

The strategy of casting all these different possible interpretations may backfire, however. First, some of these interpretations bring economic models closer to ethics or political philosophy than to models in the domain of empirical science. The distinction between positive, normative, and analytical interpretation of models is illuminating in this sense. With normative interpretation, the authors mostly refer to the prescriptive uses of models in giving, e.g. policy advice, or being useful in mechanism/institution design. But they also relate normative issues concerning how ‘economic research should be conducted’ (p. 3). GPSS pay more attention to analytical interpretations, however. The authors compare an analytically interpreted model to an architectural maquette of a town square ‘testing the feasibility of a possible square’ (GPSS, p. 4). Indeed, some of the examples presented, such as Aumann’s 1987 *Econometrica* article on correlated equilibrium or Arrow’s famous impossibility theorem, appear to be of this nature. We do not deny the importance of these contributions and do admit the value of methodological contributions that help clarify concepts and the relations between them.^9^ Nevertheless, if many or most models in a discipline are interpreted as ‘analytical’, one may feel tempted to classify the discipline as regressive.

The authors correctly observe that interpretations of models are shifting over time. However, as already discussed in the previous section, most of the examples they give contain a shift from positive interpretations to normative and especially to analytical interpretations: Savage started to interpret expected utility as a normative theory, reputational models ‘could better be viewed as tests of consistency rather than positive explanations’ and, furthermore, ‘models of backward induction and the common knowledge of rationality are more appropriately viewed as analytical models’ (GPSS, p. 13). Gone are the days of ‘bold conjectures and refutations’. Instead, if a model does not stand up to empirical scrutiny, a theorist can always claim that it has normative validity and if they cannot even say that, there is always the analytical option of arguing that a model is merely a proof of concept.^10^

Second, and importantly, the route into interpreting models as analytical tools gives empirically oriented researchers the excuse not to take economic theory seriously and therefore not to take these models as a basis for their analysis, as they are descriptively adequate anyway. Consequently, it is not surprising that the gap between economic theory and empirical research is widening. Such a development creates more scope for empirical research that is more loosely, or hardly at all, based on economic theory, and for theoretical research that is only speaking to the relatively small community of fellow theorists. Important feedback between theoretical and empirical research is getting lost in economics. The move towards interpreting economic models as only having normative or analytical purposes may also be a reason for the decreasing esteem of microeconomic theory in economics.

## 6. Economic modelling reconsidered

GPSS appear to play two tunes at once. On the one hand, they claim to be engaged in sociology of science, but in emphasising the interpretative flexibility of economic models they are in fact *defending* microeconomic theory as it has been practiced. They portray economic models as analytical and methodological exercises but still appear to think that economic models can depict some causal relations, capturing some essential features of some economic situations. We assume that many theoretical economists in fact agree with them. Yet, it’s hard to ignore the apologetic tone of GPSS’ paper. According to their narrative, as time has passed, several celebrated models, originally proposed with ‘positive’ interpretation, only qualify for a normative or an analytical interpretation. Consequently, the biographies of many economic models appear disappointing, progressing from youthful vigour, early empirical promise, and great expectations to somewhat disillusioned maturity, with some technical tricks in reserve. Is this all that economic models can do? Does not the attempt of GPSS to immunise economic models from empirical critique make them eventually irrelevant for scientific research and economic policy? Indeed, we do not think that defending economic models by mainly appealing to their analytical or methodological function is a recommendable, or unavoidable, strategy.

From the perspective of contemporary philosophy of science, the ease with which GPSS switch from ‘positive’ interpretations to analytical and methodological ones is surprising. It is as if they took the criticism concerning the unrealistic model assumptions and results at face value. Such an interpretation is in no way inevitable. The problem of unrealistic assumptions is a traditional philosophical problem concerning scientific modelling, usually discussed in terms of the notion of idealisation. Importantly, the problem of idealisation does not just concern economics. The ‘economic exceptionalism’ GPSS portray does not get support in this regard. For instance, in biological sciences, and not just in evolutionary game theory, modelling has been criticised precisely for employing highly unrealistic assumptions (Kingsland, 1985). Both economics and biology study complex multi-layered systems, often with agents/individuals, and without general principles that would allow for accurate prediction and measurement.

There are several theories of idealisation (Weisberg, 2013). Some of them consider idealisations to be deficiencies of models, typically related to the needs of tractability and computability, and as such, in principle corrigible by theoretical and methodological advances (McMullin, 1985). Others consider idealisations epistemically beneficial in that they focus on the causal difference makers, or, more modestly, isolate causal contributions of some causal factors of interest (Cartwright, 1999; Mäki, 1992). Recently, Rice (2018) and Carrillo and Knuuttila (2022) have argued that many idealisations are holistic, relating to the mathematical and other formal methods used that are crucial for the integration of models and cashing out their outcomes. While Rice still approaches idealisations traditionally as distortions, Carrillo and Knuuttila (2022) argue that idealisations can often only be understood in relation to the conceptual and representational tools exploited in model building, and in relation to the research programmes that such tools and models define. It is fair to conclude that in scientific modelling, all these types of idealisations are at play, and so, unrealistic assumptions do not in and of themselves rob a model of the ability to convey insights on important features of economic phenomena.

Why is it then that GPSS so readily give economic models an analytical/ methodological reading, without considering in more detail the epistemic roles and functions of different assumptions in economic modelling? More generally, GPSS seem to assume that for a model to be a ‘positive’ model, it would need to depict economic systems accurately, at least in some respects and to a certain degree. Indeed, much of philosophical discussion likewise ties the epistemic value of models to more or less accurate representation. Several recent philosophical accounts do not make such assumptions, however. These approaches view models as investigative instruments (Morgan & Morrison, 1999; Morgan, 2012), epistemic artefacts (Knuuttila, 2011, 2021), exemplifications (Elgin, 2004), or fictions (Frigg, 2010; Suarez, 2008). The main idea is that modelers learn by constructing models that are often designed to answer some empirical or theoretical questions (the latter being often, but not always, motivated by empirical observations). Consequently, a key to the epistemic value of a model is not just the relationship of the model to the world, but the analysis of the world depicted in the model (Morgan, 2012), which enables the modeller to study the possible dependencies between various economic factors and variables.

Does not the model eventually need to adequately represent economic systems to be able to give any knowledge of them? And if it does not, is it not then merely a useless object? Such a conclusion is too hasty. First, many unrealistic assumptions in modelling are due to the needs of mathematisation (Boumans, 2005). The constraints introduced by mathematical, statistical, and computational methods are both delimiting and enabling when it comes to modelling practice. A realistic mathematical model would not likely be tractable, and even simple models are able to give some insight on possible economic dependencies. Second, and related to the previous point, especially theoretical models are modal in nature: they study possibilities, impossibilities, necessities, and contingencies. Third, the extent to which the dependencies studied by a model hold in the actual economies is a matter of extended inquiry and not a question of some uniquely determinable representational relation between a single model and an economic ‘target’ system. To require that the assumptions of a model were realistic in some transparent fashion, or at least some of them were, is simplistic. Any model is part of a fabric of various kinds of epistemic activities and instruments covering theories and other theoretical models, observations, statistical and computational analyses, and experiments: the justification of a model is distributed across these various epistemic activities (Knuuttila, 2011). Also, part of the justification is already built in, given the theoretical, empirical, and conceptual resources and other ingredients needed in model-building (Boumans, 1999). In view of such an extended justificatory network, the strategy of protecting economic theory by selectively introducing different interpretations is counterproductive.

In our view, GPSS’s idea of viewing economic models as theoretical cases has the potential of giving them a more ‘positive’ role. As we have argued above, modelling can be approached as a distinct theoretical strategy that addresses different types of situations applicable to various problems and domains. This seems to be what GPSS have in mind but do not fully articulate when underlining the importance of model-based understanding when encountering novel problems that require theorists ‘to project from one domain [of economics] to another’ (GPSS, p. 8). Perhaps due to their representationalist premises, they fall back on considering theoretical cases analytical or methodological exercises. For instance, they claim that the Akerlof lemons model is a methodological model highlighting the importance of asymmetric information. Akerlof’s model has clearly been pivotal in introducing modelling as a new style of theorising. However, as pointed out by Sugden (2000), considering the lemons model as a simplified representation of a market for used cars is beside the point. The model is a simple system constructed to study the effects of asymmetric information, and its implications reach far beyond the sale of used cars to any kinds of markets with asymmetric information: that is, it is modelling a kind of situation that can then be applied to particular cases (either empirical or lightly characterised). But as we have already pointed out above, this should be clear to anyone reading Akerlof’s original paper: the first paragraph talks about the labour market, underdeveloped countries, money, and the notion of insurability, while the market for automobiles is only introduced as an example in the next section. In addition, the second paragraph of his introduction already mentions that in markets with information asymmetries ‘social and private returns differ, and therefore, in some cases, governmental intervention may increase the welfare of all parties’ (Akerlof, 1970, p. 488). Thus, Akerlof (1970) did not only seek to make a methodological contribution. He pointed to the fact that a very diverse set of markets can all share a fundamental feature, namely that one side has more accurate information than the other side and that this creates a mechanism whereby inefficiencies arise, as not all mutually beneficial trade is realised. The notion of a theoretical case that can be taken to represent a common kind of situation found in the economy, allows for a ‘positive’ interpretation of Akerlof’s market for lemons. GPSS would not need to regard it as an example of ‘models that were initially presented as ‘straightforward’ predictive science [and] can find themselves re-interpreted as methodological contributions’ (GPSS, p. 13).

Let us conclude with a casual sociology of science observation. In the field of industrial organisation, a field that used to be crucial for understanding the functioning of markets – itself a key area of economics research – seems to be moving more and more towards business schools outside of economics departments, and to top journals in management and marketing, such as *Management Science* and *Marketing Science*.^11^ The reason we mention this is that this development may not be unrelated to the divergence between theoretical and empirical research in economics. In more applied areas, such as industrial organisation, theoretical models are meant to be inspired by empirical observations and so, to provide understanding of the real world behaviour of firms, to explore new mechanisms that may be at play, and inspire empirical research into uncovering whether these hypothetical mechanisms are really at play. These models may not be ‘deep’ from a mathematical point of view and neither practical enough from a firm or policy perspective, but they are important nevertheless in helping to think about firms’ incentives and how they interact in markets. Business schools and management and marketing journals realise this, creating an environment where these theoretical insights can flourish in relation to actual policy discussions and empirical research, whereas in some other areas of economics, the divide between theoretical and empirical research continues to widen.

## Notes

1 See, e.g. https://www.nobelprize.org/prizes/economic-sciences/2019/

2 See, e.g. https://www.nobelprize.org/prizes/economic-sciences/2021/

3 Sugden (2000) sees these as good examples of what the category of ‘theoretical models’ can achieve.

4 The other four distinctions are (i) predictive versus explanatory tasks of models; (ii) rule-based versus case-based models; (iii) division between economic models, economic methods, and economic methodology; and (iv) the uses of models in prediction versus in critique. Three of the distinctions are based on the earlier articles of GPSS.

5 It is not clear to us what is supposed to be the difference between the two, though GPSS also mention that an analytical paper *–* not an analytical interpretation of a model – is more convincing if it makes a methodological contribution.

6 From a philosophical perspective, GPSS’s notion of explanation is rather idiosyncratic. They meld explanation together with understanding, while attributing to understanding ‘a-ha’ moments, neither of which corresponds to how these notions are discussed in the philosophical literature (see e.g. Regt, 2017; Ylikoski, 2009). Such philosophically awkward formulations do not concern us here, however. Economists are not expected to be philosophers, and vice versa, though we think that some parts of GPSS’s argumentation could have been strengthened with some guidance from philosophical discussion.

7 While in philosophy of science analogical reasoning often refers to a transfer of a model from one discipline to another (e.g. Grüne-Yanoff, 2011; Knuuttila & Loettgers, 2020), GPSS discuss an application of a model – or rather a model template (Knuuttila & Loettgers, 2023) – to different domains within economics, e.g. applying supply and demand models from product markets to the labour market, or market for children, etc.

8 Such multiple modelling is frequently approached in philosophical discussions through the notion of robustness analysis (e.g. Kuorikoski et al., 2010). Ylikoski and Aydinonat (2014) discuss multiple modelling in terms of a cluster of models providing a menu of possible explanations. Recent philosophical discussion has addressed the cross-domain and cross-disciplinary application of some models in casting them as model templates (e.g. Knuuttila & Loettgers, 2023).

9 This is akin to Kuhn’s (1962) puzzle solving nature of normal science.

10 Quite a few contributions of mathematicians to economic theory seem to be similar to solving puzzles like ‘moving 3 match sticks to get the coin inside the house’, where at the start of the puzzle, there is a coin placed outside the shape of a house outlined using 5 match sticks. The assignment is to move 3 of the matches so that the coin is placed within the house. Solving puzzles like that may show creativity and ingenuity, but if too much effort of a discipline goes into this direction, the field becomes increasingly devoid of empirical relevance.

11 For a few (non-exhaustive) references of theoretical industrial organisation economists publishing in management and marketing journals, see e.g. Alexandrov & Bedre-Defolie, 2014; Anderson & Renault, 2013; Bar-Isaac & Shelegia, 2023; Garcia et al., 2023.
